# MAILED SURVEYS WITH NONAGENARIANS - CHALLENGES AND LESSONS FROM THE VITALITY 90+ STUDY

**DOI:** 10.1093/geroni/igz038.3184

**Published:** 2019-11-08

**Authors:** Marja Jylha, Jani Raitanen, Kristina Tiainen, Pauliina Halonen, Linda Enroth

**Affiliations:** Tampere University, Gerontology Research Center, Tampere, Finland

## Abstract

Reliable population-based data on health, functioning and quality of life among very old people are scarce because only during the last decades this age group has grown to be an important segment of population, and because data collection among the oldest old is challenging. Due to poor health, problems in hearing and vision, cognitive decline, and institutionalization, very old individuals may not be able to participate in research studies, or, the information they give may not be reliable. In the Vitality 90+ Study, the whole population aged 90+ in the Tampere area, Finland, has been investigated six times since 2001. Mailed surveys have been conducted in years 1995, 1996, 1998, 2001, 2003, 2007, 2010, 2014, and 2018. In each data collection, the response rate has been ca 80%. The questionnaires and the wording of the questions have been identical in each survey round, which provides data for investigating time trends in health, functioning, and quality of life. Linkages with national population and care registers are used for studying mortality and care use. In this poster, we analyze the impact of 1) exhaustive base data, 2) the questionnaire, 3) including institutionalized individuals and proxy answers, on the findings and on the quality and reliability of the data. We conclude that mailed surveys can be a feasible method of data collection among very old people, but only in favorable local circumstances and with great efforts from the research group.

